# Coronavirus infection (SARS-CoV-2) in obesity and diabetes comorbidities: is heat shock response determinant for the disease complications?

**DOI:** 10.1186/s13098-020-00572-w

**Published:** 2020-07-16

**Authors:** Mauricio Krause, Fernando Gerchman, Rogério Friedman

**Affiliations:** 1 Laboratory of Inflammation, Metabolism and Exercise Research (LAPIMEX) and Laboratory of Cellular Physiology, Department of Physiology, Institute of Basic Health Sciences, Universidade Federal do Rio Grande do Sul, Porto Alegre, RS Brazil; 2 Endocrine and Metabolic Unit, Hospital de Clinicas de Porto Alegre, Porto Alegre, RS Brazil; 3 Graduate Program in Medical Sciences: Endocrinology, Department of Internal Medicine, Faculty of Medicine, Porto Alegre, Brazil

**Keywords:** SARS-CoV-2, Inflammation, Heat shock response, Metabolic diseases

## Abstract

Chronic inflammation is involved in the pathogenesis of several metabolic diseases, such as obesity and type 2 diabetes mellitus (T2DM). With the recent worldwide outbreak of coronavirus disease (SARS-CoV-2), it has been observed that individuals with these metabolic diseases are more likely to develop complications, increasing the severity of the disease and a poorer outcome. Coronavirus infection leads to the activation of adaptive and innate immune responses, resulting in massive inflammation (to so called *cytokine storm*), which in turn can lead to damage to various tissues, septic shock and multiple organ failure. Recent evidence suggests that the common link between metabolic diseases and SARS-CoV-2 is the inflammatory response (chronic/low-grade for metabolic diseases and acute/intense in coronavirus infection). However, the ability of the infected individuals to resolve the inflammation has not yet been explored. The heat shock response (HSR), an important anti-inflammatory pathway, is reduced in patients with metabolic diseases and, consequently, may impair inflammation resolution and control in patients with SARS-CoV-2, thus enabling its amplification and propagation through all tissues. Herein, we present a new hypothesis that aims to explain the increased severity of SARS-CoV-2 infection in people with metabolic diseases, and the possible benefits of HSR-inducing therapies to improve the inflammatory profile in these patients.

## Background

COVID-19 (Coronavirus Disease-2019), a disease caused by SARS-CoV-2 (Severe Acute Respiratory Syndrome-Coronavirus-2), was identified in China in January 2020, after notification of a series of pneumonia cases of unknown cause [1, 2]. Currently (May 2020), over 3.7 million cases have been identified worldwide, with more than 260 thousand registered deaths. The clinical manifestations of the disease range from asymptomatic to severe viral pneumonia with respiratory failure and death.

The most frequent complications of the disease are acute respiratory distress syndrome (ARDS), heart failure, septic shock and/or multiple organ failure [3–5]. Risk factors associated with a higher probability of hospitalization and higher mortality are advanced age and the presence of chronic conditions such as hypertension, diabetes, cardiovascular disease [3, 4] and, possibly, obesity [6]. In the case of diabetic subjects, the higher susceptibility may be related to impaired immune response caused by the chronic metabolic dysfunction [7–9]. Interestingly, a common hallmark of the described risk factors is the insulin resistant state [10].

Coronavirus infection results in activation of adaptive and innate immune responses, resulting in massive inflammation—referred to as “*cytokine storm*”—which can lead to various tissues’ damage, locally and systemically, in addition to lymphopenia. Recent evidence suggests that the common link between metabolic diseases and SARS-CoV-2 is the inflammatory state, and the inability to induce its resolution. Elevated levels of inflammatory cytokines, especially interleukin-6 (IL-6) [11], have been reported in patients with COVID-19, and, for this reason, anti-inflammatory therapies, such as IL-6 blockers and interleukin-1 blockers (IL- 1β) are beeing tried as a treatment.

SARS-CoV-2 infected patients can develop a delayed exacerbated immune response that contribute to the cytokine storm and tissue injury, demonstrating a role of persistent inflammation in COVID-19 infection and complications. The continuous activation of inflammatory cells, even following viral clearance, appears to involve TLR-7 signaling overactivity and the perpetuation of exaggerate inflammation [12, 13]. Here, we wish to discuss, in addition to the well accepted knowledge on persistent inflammatory response in COVID-19 infection, a not well explored pathway, looking at the resolution of inflammation, the heat shock response (HSR).

The control of the inflammatory response depends, of course, on its mediators (cytokines, LPS, among others), but also on a series of programmed mechanisms for resolving inflammation, such as the heat shock response pathway. This molecular pathway is required for physiological adjustments in proteostasis and normal stress adaptation [14]. Recent evidence has shown that the HSR pathway, an important inflammation resolution pathway, is reduced in patients with metabolic disease [15]. Consequently, appropriate resolution of inflammation is impaired, allowing its amplification and propagation through all tissues, which, in patients with SARS-CoV-2 substantially increases the rate of complications [15–17].

## Hyperinflammation in COVID-19 infection, the heat shock response, and the resolution of inflammation

Critical SARS-CoV-2 patients share common features such as lymphopenia, hypercoagulability and a hyperinflammatory syndrome named “cytokine storm” (elevation in IL-6, CRP, TNF, MCP1, IL-1β levels and others) [18]. This uncontrolled release of cytokines can rapidly evolve to septic shock and multiple organ failure. In fact, elevated levels of IL-6 were found to be a stable indicator of poor outcome in patients with severe COVID-19 with pneumonia and ARDS [19]. Thus, it would be desirable to identify and treat the hyperinflammation using approved (and safe) therapies, to reduce organ damage and mortality [19]. To date, several potential anti-inflammatory therapies are under scrutiny, including glucocorticoids, IL-6 antagonists and JAK inhibitors [18].

Another candidate approach to alleviate COVID-19-related immunopathology, still not well explored, may involve the other side of the inflammatory response: the resolution of inflammation. Resolution of inflammation starts soon after the first inflammatory signals and leads to local and/or systemic elevation of temperature, triggering a conserved response of a transcriptional program based on the activation of heat shock transcription factor-1 (HSF1), the *heat shock response* (HSR) [20]. HSF1 activation initiates the machinery for the rapid production of the anti-inflammatory and cytoprotective heat shock proteins (HSP), of which the most sensitive and expressed is the 70 kDa family of HSP (HSP70), in addition to other small heat shock proteins [21, 22].

The HSR (thus HSP70 expression) is essential to protect the cells against a wide range of non-lethal stresses, such as oxidative, thermal, exertional, ischemic, metabolic and others [17]. Its content is increased up to 2% of the total cellular protein during stress [23]. HSP70 (encoded by the HSPA1A gene in humans), is a classical molecular chaperone that interacts with other proteins (unfolded, in non-native state and/or stress-denatured conformations), avoiding inappropriate interactions, formation of protein aggregates and degradation of damaged proteins, as well as helping the correct refolding of nascent proteins [24]. In addition to its several functions (anti-apoptosis, protein translocation, metabolism, and others) [17], this protein exerts, intracellularly, a potent anti-inflammatory effect [25]. The anti-inflammatory effect of HSP70 is mainly attributed to its capacity of interaction with NF-κB, decreasing its activity [26]. HSP70 is able to associate with the complex formed by NF-κB with its inhibitor (IκB), stabilizing this complex and thus impeding NF-κB translocation to the nucleus [26]. NF-κB activation is particularly involved on the mechanisms of insulin resistance through the induction of several inflammatory proteins, such as iNOS and NAPDH oxidase, inducing nitrogen and oxygen radical species formation and the consequent blockage of insulin cascade [25, 27]. Thus, HSP70-mediated NF-κB inhibition can also ameliorate insulin sensitivity [17].

While iHSP70 has anti-inflammatory effect, on the other hand, when released to the extracellular environment (eHSP72), this protein exerts opposite effects, inducing inflammation and immune activation. Extracellular HSP72 is receiving more attention since its regulatory role on immune cells are still under discussion. This protein can be released by different cells and stress conditions such as acute exercise and heat [15] and is involved in several conditions such as insulin resistance and acute lymphoblastic leukemia [28]. The effects of eHSP72 are still under debate since pro and anti-inflammatory results were described. For example, extracellular HSP72 negatively regulates the acute inflammatory cytokine synthesis by monocytes through the activation of HSF-1 to the inflammatory gene promoters [29, 30]. On the other hand, eHSP72 may bind to TLR2 and 4, activating innate immune responses which may lead to adaptive immune responses through the activation of NF-κB and JNK by a pathway related to IL-1 receptor-associated kinase (IRAK) family of protein kinases [31]. In addition, eHSP72 may induce a direct anti-inflammatory response through a TLR2-ERK-STAT3-IL-10 dependent pathway [32]. The final effect may be dependent on the ratio between iHSP72/eHSP72, as we recently suggested [17]. Until now, no report study the effects of SARS-CoV-2 on eHSP72.

**Fig. 1:**
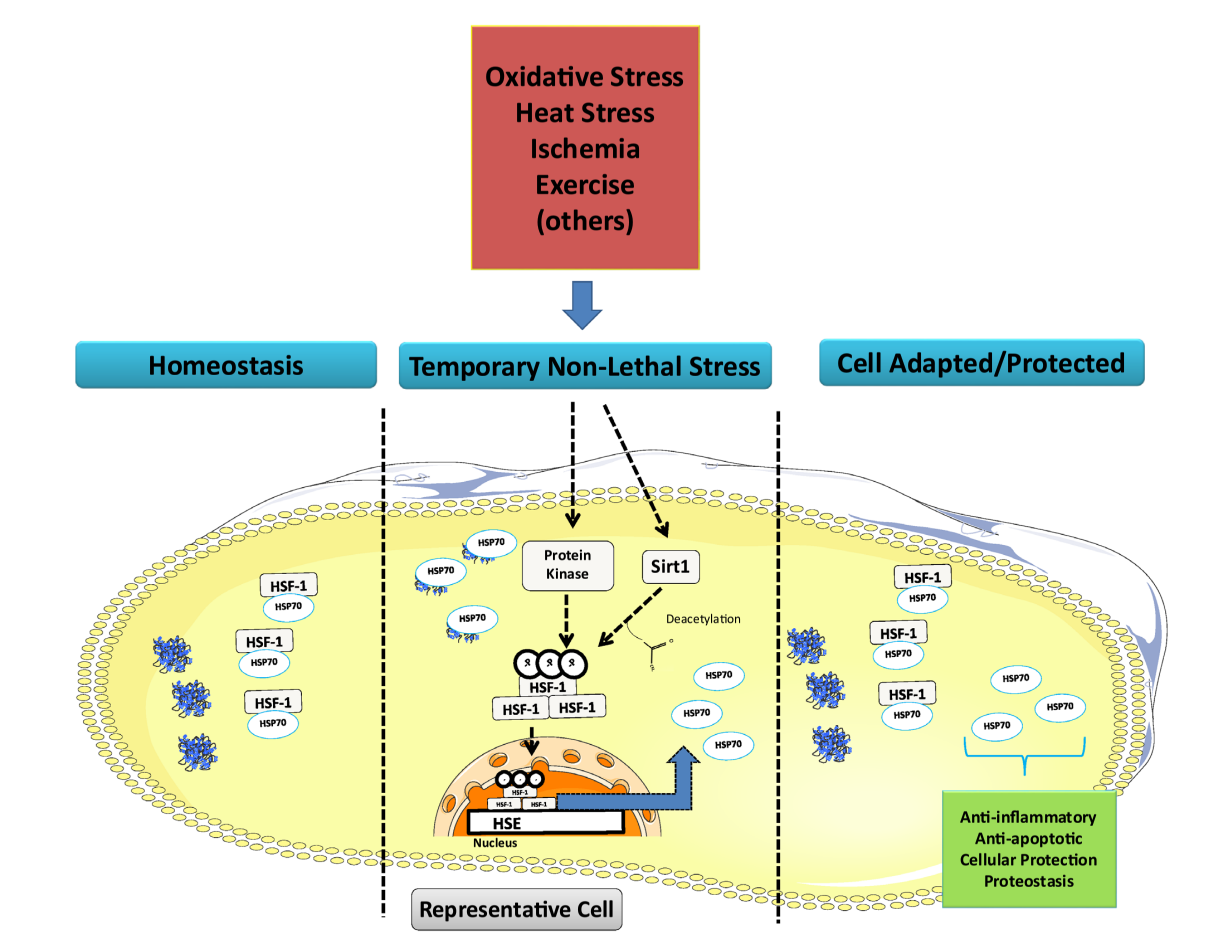
Heat shock response and HSP70 function. The Activation of the heat shock response after non-lethal stress. (I) At rest HSF-1 is inactive in a monomeric state bonded with the cytosolic HSP70s, located in the cytosol. P: Functional Proteins. (II) Under stress conditions and in the presence of denatured proteins (DP), HSP70 releases HSF-1 and subsequently binds to denatured proteins, acting as chaperones (aiding protein refolding) and releasing HSF. Serine-phosphorylation and trimerisation of HSF-1 induces enhanced HSF-1 DNA binding affinity. The binding of the trimeric HSF to HSE initiates the transcription of the HSP mRNA. Additionally, SIRT1 prolongs HSF1 binding to the promoters of heat shock genes by maintaining HSF1 in a deacetylated form. (III) After recovery from stress, HSP70 rebinds to HSF-1 so exerting an inhibitory effect on HSF-1/HSE binding. Overall, stress adaptation is associated with increased levels of HSP70

A detailed description of HSR is available elsewhere [25], and involves several key modulators such as NAD^+^-dependent deacetylase sirtuin-1 (SIRT1). Figure 1 summarizes the HSR and the production of HSP72. As briefly described, stress-activated HSF1 leads to a loop of positive feedback that provides a robust anti-inflammatory response. However, a mandatory pathway to maintain a normal chaperone machinery (HSR) is insulin signalling [17]. Hampered insulin signalling will lead to a deficient ability to induce HSR and the resolution of inflammation. Obese, insulin resistant individuals, and elderly people (with insulin resistance) have been found to have lower levels of HSP70 [15, 33, 34]. Not surprisingly, obesity-related, chronic inflammatory states show depressed HSR [15, 35, 36]. Thus, a lower HSR in insulin resistant individuals might be, at least in part, responsible for the exacerbated levels of inflammation and the worse prognosis observed in those infected by SARS-CoV-2, in comparison with insulin sensitive subjects.

## Heat shock response in insulin resistant subjects: the role of chronic inflammatory-related conditions

Inflammation is a common feature in elderly, obese and diabetic subjects. In comparison to acute inflammation (in response to injury or infection), in chronic conditions the levels of inflammatory mediators are lower, but remain chronically elevated, a situation called low-grade inflammation. The origin of low-grade inflammation is multifactorial. Evidence suggests that the inflammatory profile is directly connected with the unfavourable changes in body composition [33, 37]. For example, the expansion of the adipose tissue (specially visceral) leads to increased recruitment of blood monocytes and their polarization to inflammatory cells (M1 phenotype) [14].

Adipose tissue expansion results in the release of several cytokines, such as TNF-α [38], leading to the activation of serine threonine kinases, JNK (c-jun amino terminal kinase), and the inhibitor of IκB, IKK (IκB kinase kinase) [34]. Both JNK and IKK phosphorylate IRS-1 on Ser-307, leading to inactivation of the insulin receptor [34], and, eventually, to insulin resistance [10]. In addition, TNF-α signalling also promotes the activation of proteins and enzymes that initiate the massive production of free radicals, reactive oxygen species (ROS) and nitrogen reactive species (RNS) [39], all connected to insulin signalling impairment [39].

As previously mentioned, individuals with insulin resistance may present a blunted HSR (thus reduced HSP72 expression), and an insufficient ability to resolve inflammation. But what is the convergence point between insulin signalling and the HSR pathway? One key point is the suppression of HSF-1 activation and binding to HSEs (heat shock elements), via an increased activity of the enzyme glycogen synthase kinase-3β (GSK-3), and decreased HSF1 expression [40]. HSF-1 is negatively regulated by GSK3β, a serine/threonine kinase that phosphorylates this factor on Ser303, keeping it in its inactive form in the cytosol. Insulin action is mandatory for the inhibition of GSK-3β activity. Therefore, depressed insulin signaling results in the maintenance of GSK-3β function, and chronic inhibition of HSF1 activation, leading to lower HSR in insulin resistant individuals. In fact, lower expression of HSF1 has been identified in subjects with metabolic diseases [15, 33, 36].

In addition, low-grade inflammation (present in many obese, diabetic and elderly people) can inhibit HSR at gene regulatory level: i) TNFα may transiently repress HSF1 activation [41] and ii) JNK1 can phosphorylate HSF1 in its regulatory domain causing its suppression [42]. The opposite regulation is also demonstrated since the promoter region of TNFα gene contains an HSF1 binding site that represses TNFα transcription, and thus loss of this repressor results in sustained production of TNFα [43] and increased susceptibility to endotoxin challenge [44]. These data may explain why the induction of HSP72 reduces the expression of inflammatory genes such as TNFα, IL- 1, IL-12, IL-10, and IL-18 [45].

**Fig. 2:**
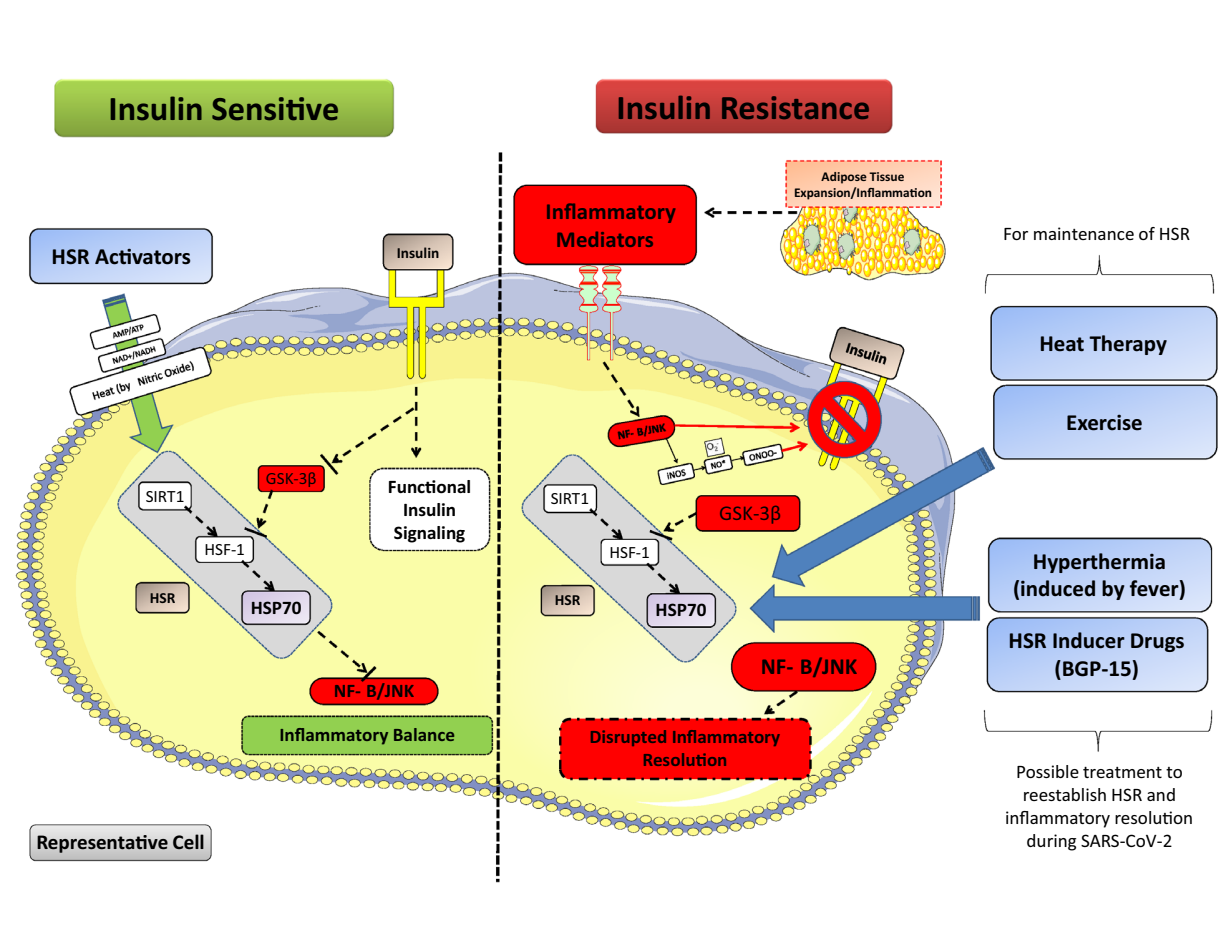
Heat shock response in healthy and in insulin resistant state. In insulin sensitive state, activation of insulin signalling will lead in inhibition of the enzyme GSK-3β (by phosphorylation). In this case, activation of HSR, when stimulated, is normal and HSP72 can maintain NF-κB inhibition, thus an inflammatory balance. Obesity (adipose tissue expansion) and physical inactivity initiates a chronic low-grade inflammation that spread to all tissues. The inflammatory mediators (cytokines, TLR ligands and others) can induce the activation of NF-κB and JNK, leading to ROS/RNS overproduction (by increase activity and expression of inflammatory enzymes) and inhibition of insulin signalling. In the presence of insulin resistance, GSK-3β become activated and inhibits HSF1 activity and expression, resulting in a blunted HSR. Under this circumstance, no inhibition over NF-κB results in amplification of inflammation and no resolution, causing a vicious inflammatory cycle. Heat therapy (hot water immersion or sauna) and exercise can activate HSR and ameliorates insulin signalling and inflammation. Two potential alternative therapies that may be applied to restore HSR and reduce inflammation in SARS-CoV-2 infected patients is the rationale use of antipyretic drugs (allowing increases in temperature, thus improving HSR) and the use of HSR activator drugs, such as the BGP-15

In summary, low-grade inflammation, found in elderly, obese or diabetic subjects, induces (through the release of inflammatory cytokines) inhibition of insulin signaling and chronic activation of GSK-3β, reducing HSF1 activity and blunted HSR (Fig. 2). Therefore, it seems reasonable to expect that patients with these profiles, when infected by SARS-CoV-2, would be unable to activate the resolution of the inflammation, giving way to a vicious cycle of inflammation that might increase the probability of complications found in these populations. It is not surprising that diabetes significantly increases the risk of Covid-19 progression and the rates of mortality [46]. Despite the fact that not all obese and old patients who progress to severe COVID-19 complications have a formal previous diagnosis of diabetes, both aging and obesity are associated with low-grade inflammation, and some degree of insulin resistance.

Regarding gender differences, considering that infected men are more at risk than women, gender-specificity of HSR needs some consideration. Again, there is a significant lack of data. Under the light of current knowledge, we could speculate that the observed gender differences in risk may be related to the higher levels of estrogen (E2) in the female subjects. E2 is known to protect women from insulin resistance, and insulin signaling is mandatory for normal HSR) [47, 48]. E2 is related to maintenance of a normal HSR (thus HSP72 expression) [49, 50]. Therefore, the hormonal profile of female patients might perhaps explain not only the different clinical outcomes but it could also offer some insight into the mechanisms behind this difference.

## Strategies to increase the HSR in SARS-CoV-2

Different strategies have been used to increase the HSR and raise the levels of HSP72 in patients with inflammatory-related diseases—such as cardiovascular, metabolic and neurodegenerative diseases [17]. Among the alternatives, several data have shown the efficacy of exercise training [15], heat therapy (hot water immersion, sauna or hot tub) [51], pharmacological agents (such as BGP-15, a hydroxylamine derivative) [52], and finally, the rational use of antipyretics for fever control.

Regarding exercise, it is expected that individuals engaged in exercise training, present higher levels of HSP72 (efficient HSR machinery). In fact, elevated levels of HSP72 have being associated with resistance to metabolic diseases (such as diabetes), increased cardiovascular fitness (higher oxygen consumption, VO_2max_) and reduced inflammation [53]. These mechanisms give support the hypothesis that active individuals (with better cardiorespiratory fitness) could be more resistant to the inflammatory effects of COVID-19 infection, as recently suggested [54].

Other alternative intervention to increase HSR is heat therapy. It consists in inducing a passive elevation of the central body temperature, using hot water immersion or sauna. The benefits of heat therapy in metabolic diseases have been studied and confirmed [51]. However, in the setting of COVID-19, these two interventions cannot be applied.

Considering that HSR is activated by elevation of body temperature, another potential strategy to improve HSR involves the rational use of antipyretics in infected patients. In fact, the inflammatory response, triggered by any infection (including SARS-CoV-2), will lead to the activation of the nuclear factor NF-kB, the master regulator of inducible production of cytokines and inflammatory enzymes. This culminates into the release of several factors (such as prostaglandins), capable of inducing fever (hyperthermia), and in turn activating HSR and aiding the resolution of inflammation [26]. Thus, fever is important for the HSR induction. This should be taken into account when using antipyretic drugs to control fever. Lowering body temperature, down to normal levels, with antipyretics, may potentially hamper the natural HSR and the resolution of inflammation. This has not been properly tested to date. The challenge, of course, is to find an optimal temperature that still allows HSP72 to increase, without causing any tissue heat damage.

Finally, the use of pharmacological agents may provide a strategy to improve HSR in patients with metabolic diseases. A candidate drug is BGP-15. BGP-15 is a pharmacological inducer of HSP72 that has been shown to be safe and well tolerated in Phase II clinical trials in patients with diabetes and insulin resistance [55, 56]. The use of BGP-15 in animal models was found to induce metabolic benefits, besides reducing inflammatory signaling and improving respiratory muscles during mechanical ventilation [52].

## How to measure the heat shock response?

There are several technical ways to determine the HSR in biological samples. The majority of studies use the quantification of gene and/or protein levels of factors (or their products) involved in this pathway, such as HSF1, SIRT1, HSP72, and other chaperones. However, with this strategy, only baseline levels can be measured, leading, sometimes, to divergent results. For this reason, we suggest the use a “heat stress test” to determine the real chaperone machinery capacity of the cells to express HSP72 (and release, in the case of peripheral blood mononuclear cells) in response to a heat challenge [15].

**Fig. 3:**
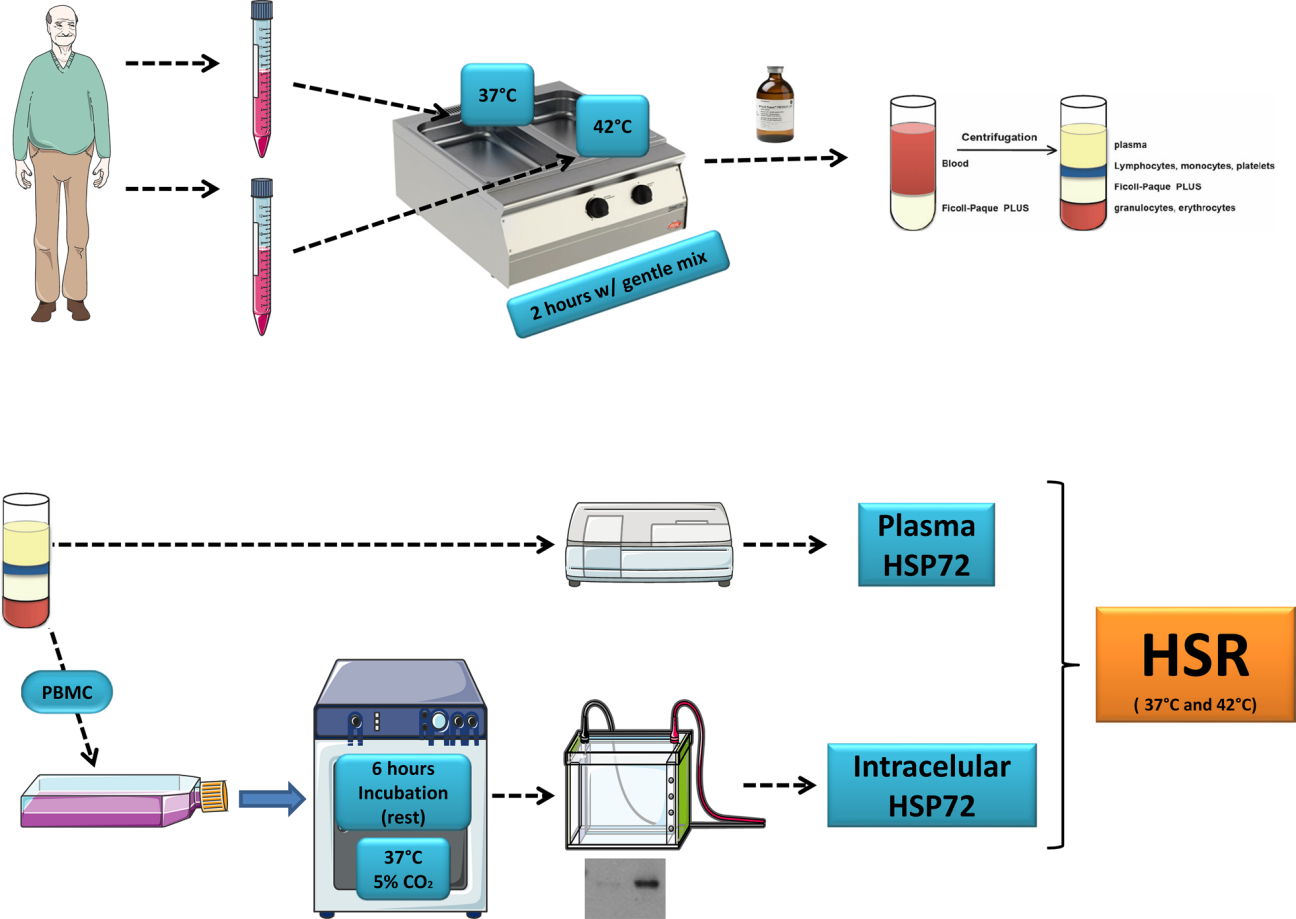
Heat shock response test in blood of SARS-CoV-2 patients. After harvesting, whole blood is immediately incubated at two different temperatures: 37 °C (control) and 42 °C (heat stressed) for 2 h in water bath (with gentle mix every 15 min). After the incubation, total blood is centrifuged to isolate plasma/serum and PBMC through density gradient separation. Then, plasma can be used for the direct analysis of extracellular HSP72 (eHSP72) while PBMC can be prepared for the measurement of intracellular (iHSP72). The PMBC must be washed and treated to ensure the absence of erythrocytes. PBMC are resuspended in RPMI 1640 medium (pH 7,4 supplemented with 2% NaHCO_3_, 10% bovine calf serum, 100 U/mL penicillin and 100 µg/mL streptomycin), seeded in a 24-well flat bottom plate (1 × 106 cells/well) and placed in an incubator for 6 h (37 °C in a 5% CO_2_), in order to recover from the HS and reach the peak of HSP70 expression. Cells are then removed from the incubator, appropriated lysed and the total content of proteins prepared for western Blot analysis. The difference between concentration at 37 °C and 42 °C is used as HSR index

As a model it is possible to test HSR in human peripheral blood mononuclear cells (PBMC), a major source of circulating HSP72 and representative of immune cell stress response [14]. These cells, in normal and optimal conditions, can express and release HSP72, under heat stress conditions. Briefly, after harvesting, whole blood is immediately incubated at two different temperatures: 37 °C (control) and 42 °C (heat stressed) for 2 h in a water bath (with a gentle mix every 15 min). After the incubation, total blood is centrifuged to isolate plasma/serum and PBMC through density gradient separation [57, 58]. Then, plasma can be used for the direct analysis of extracellular HSP72 while PBMC can be prepared for the measurement of iHSP72. The difference between concentration at 37 °C and 42 °C is used as a HSR index. The full protocol is described elsewhere [14] and is present in Fig. 3. In addition, the levels of extracellular HSP72 (plasma) may be used as a marker for inflammation and immune system control, since, as an extracellular protein, HSP72 induces inflammation through the activation of Toll-like receptors (TLR2 and 4) [17, 25].

## Conclusions and perspectives

The *cytokine storm* syndrome induced by coronavirus infection can lead to severe tissue damage and evolve to septic shock and multiple organ failure. For this reason, it is recommended that this hyperinflammation it treated to reduce the disease-related complications and mortality, especially in patients with established metabolic disease (for whom the risk is considerably higher). To date, only therapies using anti-inflammatory agents were considered. We suggest that HSR, an essential pathway for inflammation resolution, is blunted in individuals with insulin resistance and, for this reason they are at risk for complications when infected by coronavirus. This hypothesis is currently under investigation in our laboratory. Finally, we propose that the use of HSR activators should be investigated, since they could potentially alleviate the COVID-19 complications in insulin resistant patients. This may include the rational use of antipyretic drugs (to allow mild elevations of body temperature by fever, without causing heat damage, but enough to eventually lead to elevations of HSP72), and HSP72 activators such as BGP-15.

## Data Availability

Not applicable
